# Cognitive function and carotid stenosis: Review of the literature

**DOI:** 10.1590/S1980-57642012DN06030003

**Published:** 2012

**Authors:** Aurélio Pimenta Dutra

**Affiliations:** Médico Neurologista. Assessor Médico da Neurologia Vascular Fleury Medicina Diagnóstica.

**Keywords:** carotid stenosis, cognitive function, endarterectomy, stent, hypoperfusion

## Abstract

Stroke is a known cause of cognitive impairment but the relationship between asymptomatic carotid artery stenosis and cognitive function is not clear. The main risk factors for vascular disease are also related to carotid stenosis and cognitive impairment. The association of high-grade stenosis of the internal carotid artery with cognitive impairment is related to silent embolization and hypoperfusion, but it may also be present without evidence of infarction on magnetic resonance imaging. Carotid stenosis treatment may lead to a decline in cognitive function due to complications related to the procedures (endarterectomy or stenting). On the other hand, reperfusion may improve cognitive impairment. The best treatment choice is unclear, considering possible deterioration of cognitive function related to carotid artery stenosis. There is insufficient evidence to consider cognitive impairment an important factor in determining the therapy for carotid stenosis.

## Abstract (pt)

O acidente vascular cerebral é uma doença que pode levar ao declínio cognitivo, mas a relação entre a estenose carotídea e as funções cognitivas não são bem esclarecidas. Os fatores de risco para doenças vasculares estão também relacionados à estenose de carótida e ao declínio cognitivo. A associação da estenose grave de carótida interna com o declínio cognitivo está relacionada a embolia silenciosa e hipoperfusão, mas pode também estar presente sem a evidência de lesão isquêmica pela ressonância. O tratamento da estenose carotídea pode levar ao declínio cognitivo devido a complicações relacionadas aos procedimentos (endarterectomia ou *stent*). Por outro lado a reperfusão pode melhorar o declínio cognitivo. Não está claro qual a melhor decisão terapêutica considerando a possível deterioração cognitiva relacionada à estenose carotídea. Não existe evidência suficiente para considerar o declínio cognitivo um fator importante na decisão terapêutica para estenose de carótida.

## INTRODUCTION

Decline in cognition has become one of the most relevant symptoms of focus in recent years. With aging populations, the most important unmodifiable risk factor for cognitive disorders, cerebrovascular risk factors and disease, have become significant modifiable factors, particularly in the development of stroke-dementia association.^1,2^

The main risk factors for vascular disease are also related to cognitive impairment. Hypertension, diabetes mellitus, cigarette smoking, and dyslipidemia are associated with an increased risk of carotid artery disease. Some studies have suggested that stenosis of the internal carotid artery may be an independent risk factor for cognitive impairment. High-grade stenosis of the internal carotid artery may be associated with cognitive impairment even without evidence of infarction on magnetic resonance imaging and is therefore suspected as an independent risk factor for dementia.^2^ Possible mechanisms involved include silent embolization and hypoperfusion.^3^

Carotid endarterectomy (CEA) or stenting in patients with severe carotid stenosis reduces stroke risk.^2^ A possible association considering improved cognitive function and better cerebral perfusion has been hypothesized, whereas subclinical microembolic cerebral patterns occurring during revascularization may worsen cognitive function.^2^

According to population studies, some degree of carotid disease can be found in more than 75% of men and around 60% of women older than 65 years of age. Overall prevalence of over 50% carotid stenosis in the same age group is 7% in men and 5% in women. The prevalence of cognitive impairment also increases with aging.^3^

The diagnosis of advanced carotid stenosis is mostly reached in symptomatic patients based on the presence of stroke or transient ischemic attack (TIA). However, the cognitive status in these patients is often overlooked, although if cognitive impairment is present, such patients should probably be considered symptomatic.^3^

In this paper, we review some studies that have evaluated the association between cognitive decline and advanced carotid disease, possible mechanisms involved in this relationship, and how to evaluate it in clinical practice.

## EVALUATION OF COGNITIVE FUNCTION

The presence of cognitive deficit is associated to loss of one or more cognitive function. The cognitive functions are linked to different neural synapses and anatomic brain regions. In clinical practice, the Mini-Mental State Examination (MMSE) is the most commonly applied screening test and most frequently used to stratify the severity of cognitive impairment and dementia. The main problem is that the MMSE does not differentiate among cognitive functions.

Patients with severe carotid disease without previous stroke or TIA can have subtle cognitive abnormalities that are undetectable by the MMSE and may only be identified by applying specific neuropsychological tests.^4^

The use of well-defined neuropsychological tests allows the evaluation of specific cognitive functions linked to specific neural systems.^2^

These neuropsychological tests can reveal cognitive decline even in patients with carotid atherosclerosis characterized by number of plaques and total plaques, and not severe stenosis, suggesting that subclinical carotid atherosclerosis may increases the risk of cognitive decline.^5^ As an example, the Montreal Cognitive Assessment (MoCA), a more comprehensive test than the MMSE, is capable of identifying reduced cognitive status in patients with asymptomatic ICA stenosis.^6^

Transcranial Doppler may be used as a functional parameter for intracranial subclinical atherosclerotic changes, measured by the breath holding index (BHI). Performance on the BHI test is decreased and related to impaired cerebrovascular reactivity in patients with early cognitive decline.^7^

## CAROTID STENOSIS AND RELATIONSHIP WITH COGNITIVE DECLINE

The vascular risk factors (hypertension, diabetes, dyslipidemia and smoking) are also risk conditions for stroke, carotid stenosis and dementia. Theoretically, a carotid stenosis may be a direct cause of a reduced level of cognitive functioning, or it may act only as a marker of intracerebral or generalized atherosclerosis. The relationship of asymptomatic stenosis with cognitive impairment is unclear.^2^

An evaluation of the relationship between cognitive decline and atherosclerosis was carried out in the 1975 Framingham Offspring Study participants. The results suggested that internal carotid artery intima-media thickness may be a marker for cognitive impairment and be associated with higher prevalence of silent cerebral infarcts and of extensive white matter hyperintensity.^8^

The Tromsø study evaluated subjects without history of stroke and with Carotid stenosis measured by ultrasonography that showed right-sided or bilateral narrowing of ≥35%. This group was compared to subjects with and without carotid stenosis. The study demonstrated that subjects with carotid stenosis had significantly lower levels of performance on several subsets of cognitive tests.^9^

The Cardiovascular Health Study evaluated individuals with no history of stroke, transient ischemic attack (TIA) or carotid endarterectomy. Internal carotid artery stenosis was measured by duplex ultrasonography. The study found that high-grade (≥75%) stenosis of the left internal carotid artery was associated with cognitive impairment and cognitive decline during follow-up, even in participants without cerebral infarction on MRI. These observations support the notion that even asymptomatic carotid stenosis may be an independent risk factor for cognitive impairment and decline.^10^

The effect of carotid stenosis on cognitive functioning remains largely unexplained although it is believed that asymptomatic advanced carotid stenosis should be considered an independent risk factor for decline in cognitive functioning. If asymptomatic advanced carotid stenosis indeed causes cognitive impairment, the decision on surgical treatment might consider cognitive evaluation using a neuropsychological test as a clinical definition of symptomatic carotid disease and may be considered as important as the effective treatment of vascular risk factors in stroke/TIA free patients with carotid stenosis.^4^

## MECHANISM OF COGNITIVE DECLINE IN CAROTID STENOSIS

The main mechanisms related to cognitive impairment in carotid stenosis are embolization and hypoperfusion.

The presence of silent brain infarction is related to an increase risk of dementia. Silent brain infarcts may be related to carotid stenosis, embolization or hypoperfusion. They may also be present in asymptomatic carotid stenosis and are related to lacunar infarcts secondary to microangiopathy and cardiovascular risk factors.^2^

Embolization may be detected by middle cerebral artery monitoring on transcranial Doppler. A study comparing Alzheimer's disease, vascular dementia and controls, observed that spontaneous cerebral emboli were significantly more frequent in patients with both Alzheimer's disease and vascular dementia.^11^ On the other hand, the Tromsø Study and the Cardiovascular Health Study observed that cognitive impairment in patients with carotid stenosis was independent of vascular lesions observed on MRI.^9,10^

Brain hypoperfusion may contribute to the onset of clinical dementia and is observed in patients with severe heart failure and also with carotid stenosis. The Tromsø Study results showed a significant relationship between cognitive impairment and degree of carotid stenosis.^9^

## COGNITIVE FUNCTION AFTER CAROTID INTERVENTION

In most studies, cognitive function after carotid intervention has shown significant improvement. By contrast, some studies showed no change in cognitive function while other reports have noted cognitive decline due to surgical procedures.

The effect of carotid procedures on cognitive function is not fully understood but change in cognition is being increasingly recognized as an important outcome measure. A systematical literature review of 32 papers reporting on neurocognition after carotid procedures showed that assessment of cognition after carotid revascularization is probably influenced by many confounding factors such as learning effect, type of test, type of patients, control group, and lack of consensus in defining improvement or impairment after either carotid artery stenting (CAS) or carotid endarterectomy (CEA). Based on recent evidence, it is likely that carotid endarterectomy as well as carotid artery stenting do not affect neuropsychological function.^12^

Some studies have shown cognitive decline after carotid endarterectomy with mechanisms related to cerebral hyperperfusion after carotid endarterectomy, while in asymptomatic cases MRI does not always disclose structural brain damage associated with postoperative cognitive impairment.^13^ The general anesthesia carotid procedures have also been linked to early cognitive decline that is temporary in nature.^14^ Recent studies on the mechanism of cognitive decline associated with hyperperfusion have shown that cerebral hyperperfusion after CEA results in postoperative cortical neural loss that correlates with postoperative cognitive impairment even in the absence of MRI lesions.^15^ Other predictors of neurocognitive decline after CEA include advanced age, diabetes, obesity, preoperative monocyte count and presence of the APOE-ε4 allele.^16-18^

A recent study evaluated cerebral blood flow using phase contrast magnetic resonance angiography and cognitive testing preoperatively, and at 1, 6, and 12 months postoperatively. Patients with baseline impairment of MCA blood flow were more likely to experience improvement in flow after revascularization. Improvement in MCA blood flow was associated with greater cognitive improvement in attention and executive functioning.^19^

The benefit of the carotid procedure for prevention of brain infarct, expected from reduced embolism and improved hemodynamics, with regard to cognitive function remains unclear. Improvement in cognitive function following carotid reconstruction may be greater in patients with low-flow-endangered brains than in those with hemodynamically insignificant stenosis that are frequently clinically treated.^20,21^

## CONCLUSION

Carotid artery stenosis and atherosclerosis are risk factors for cognitive impairment. When there is symptomatic or severe artery stenosis, carotid interventions benefit stroke risk but may leave cognitive performance unchanged, or lead to a decline or improvement. There is no evidence to support the performance of prophylactic carotid endarterectomy or carotid stenting with the aim of preventing cognitive decline in otherwise asymptomatic patients.

The literature on cognitive outcome after carotid revascularization is complex and further studies investigating specific populations of patients with carotid stenosis will help elucidate whether carotid endarterectomy or carotid stenting is more appropriate for a given patient considering the cognitive function and risks after the procedure.

Future studies should consider standardizing neuropsychological outcomes using a uniform battery of tests across multiple centers. Studying relatively homogeneous groups of patients may help to reduce the variability inherent to cognitive studies.
